# Hydrogeophysical modeling of the groundwater aquifer units under climate variability in parts of Peninsular Malaysia: A case study of the climate-water nexus approach to sustainability

**DOI:** 10.1016/j.heliyon.2023.e13710

**Published:** 2023-02-14

**Authors:** John Stephen Kayode, Mohd Hariri Arifin, Mohd Idham Mansor, Nurul Nadia Abdul Malek, Roziah Che Musa, Sabrina Shahri, Nur Hazwani Izehar, Mohd Rozi Umor

**Affiliations:** a Department of Physics, Nigerian Army University, No 1, Gombe Road, PMB 1500, Biu, Borno State, Nigeria; b Program Geology, Department of Earth and Environmental Sciences, Universiti Kebangsaan Malaysia, 43600 UKM, Bangi, Selangor, Malaysia; c Malaysian Space Agency, No.13, Jalan Tun Ismail, 50480 Kuala Lumpur, Malaysia

**Keywords:** Climatic-water nexus sustainability, Groundwater saturation, Beranang Selangor, Peninsular Malaysia

## Abstract

Understanding of the climate-water nexus for sustainability, required good knowledge of the climate effects on groundwater aquifer units, particularly in rural communities. The studies were achieved using RES2-D modelling of the subsurface structures at the study site. Geophysical exploration with the application of 2-D Electrical Resistivity Imaging (ERI), combined with Induced Polarization (IP) method, were carried out to identify groundwater aquifers during extreme weather at *Kampung Kuala Pajam, Beranang, Selangor*, Peninsular Malaysia. The signatures obtained from geophysical explorations were used to better understand the phenomena that are responsible for groundwater depletion in the area. In recent times, there had been seasonal fluctuations in the water supply from boreholes serving the community. During the drought season, subsurface underlain this area experienced perennial acute shortages of groundwater supplies due to annual climatic variations that call for immediate solution by meeting the agricultural, domestic, and industrial water usage of the State of *Selangor*. A Pole-dipole techniques, using seven parallel lines of 400 m each at 5 m inter electrode spacing deployed to study the groundwater accumulation/aquifers within the area. Saturated groundwater occurrences zones were delineated as areas with average resistivity values of about 125 Ω-m, with corresponding chargeability of 30 ms. The methods used identified major faults along the northeast-southwest (NE-SW) directions, suitable for groundwater occurrences with approximate volume of about 2.86 Mega cubic metre (CBM), to proffer lasting solutions to the challenges being experienced by the community using a climate-water nexus sustainability.

## Highlights

• Long period of droughts occasioned by climate variations, caused declined in groundwater.
• Groundwater aquifer is greatly depleted than expected, particularly in Selangor.
• RES2-D inversion, and IP were applied to define dry-areas and groundwater aquifer zones.
• 3-D hydrogeophysical modelling of the aquifer units was used to quantify groundwater volume.
• An estimated volume of about 2.86 Mega m^3^, was obtained to proffer long-lasting solutions.

## Graphical abstract

**Figure:**
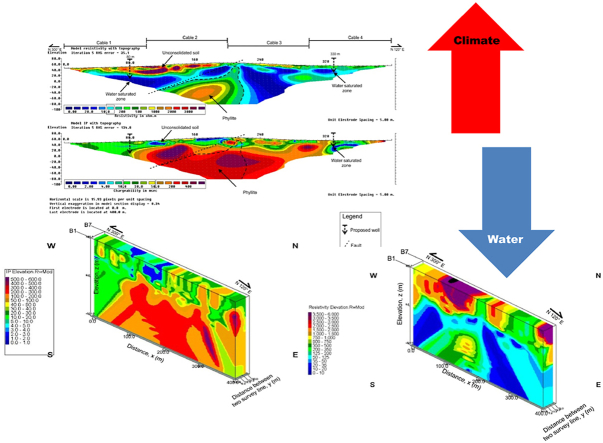

## 1 Introduction

In recent years, there had been rapid unpredictable changes in the Peninsular Malaysia's climatic conditions. The changes have been shown to adversely affects water and agricultural sustainability of most urban and rural communities in the Peninsular. Although, the Peninsular Malaysia experienced considerable amount of precipitation throughout the year during the monsoons, the country still witnessed some irregularities in inter-annual time scale precipitations [1]. Some months of extreme dryness through excessive winds and sunshine makes the country vulnerable to risks and uncertainty in water resources. To manage this unpredictable situations, adequate understanding of the interconnections between the groundwater, surface water reservoirs, climate variables as it affects the society's food security and access to hygienic water, and the need to consider, and explored subsurface groundwater resources, to compliments surface water reservoirs which suffered huge losses of water during the dried periods.

There had been perennial declined of water supply from the boreholes serving the *Kampung Kuala Pajam, Beranang, Selangor* community, particularly during the long dry season periods. The declined in the groundwater supplies may not be unconnected with the effect of climate variations occasioned by long period of droughts as recently witnessed across the Malaysian Peninsula. The rate of water shortages during the droughts and festive periods, is far higher than the recharge rate, hence, the groundwater saturation zone is much depleted than expected, particularly in the State of *Selangor*. During the El Nino Southern Oscillation, (ENSO) events, it was reported that the drought brought about by the phenomenon had greater impact on the hydrological parameters such as the dynamic earth's ecosystem, i.e., precipitation; evapotranspiration; surface water interception and runoffs; infiltration, soil moisture contents variations, river water flow, and fluctuations in groundwater storage [2]. These processes greatly affected the groundwater systems, and the hydraulic conductivity, with effect of shifting the water table, e.g. Refs. [[3], [4], [5], [6], [7]]. Groundwater level is low in this area, primarily due to adverse weather conditions, climate variability and extreme events such as, the ENSO as recently witnessed in the Malaysian Peninsula, and partly due to perennial droughts, and extensive usage during festive periods that causes colossal societal hardships and environmental problems. The foremost threat to human existence is the menace of climate variability which in most cases, affect the rural and urban community's wellbeing, social, economic, and sustainability through foods and water scarcity, e.g. Refs. [3,[8], [9], [10], [11]]. During the periods of shortfalls in terrestrial water and precipitation, the area experiences stern water stress that impacted on agricultural foods production, ecosystems, socio-economic and industrial sectors of the state.

The most critical hydrological resources responsible for nearly fifty percent of global drinking water is the groundwater. Reports by International Groundwater Resources Assessment Centre [12], have shown that roughly forty percent of agricultural irrigated water and approximately one-third of industrial water usage are extracted from the groundwater. Hence, groundwater is not meant only for consumption purposes for humans and animals’ sustainability, it supports rivers runoffs, prevents landslides and subsidence, thereby sustaining entire ecosystems [12]. Groundwater is an integral part of the climate variability, and is always pressured by global population explosion and social-economic development due to urbanization arising from the natural resource competitiveness [1]. The use of a climate-water nexus sustainability helps to understand better, the inter-connections between the groundwater resources and other essential sectors of human lives [13].

Every aspect of the planet earth is seriously devastated by the ravaging peril from the climate change which turnout to be a global concern with numerous approaches aimed at solving the challenges through sustainability. Therefore, it is highly essential to find lasting solutions or an innovative way of fighting the dreaded menace, or at best, reduce the devastating impact on the society's natural resources. Since surface water resources are generally prone to pollution which makes it highly unreliable for domestic and industrial consumptions, groundwater has become the principal source of safe, hygienic, and sustainable clean water, both for consumptions and for other uses [4,9,[14], [15], [16], [17]]. Some previous studies have been reported linking the surface runoffs after precipitation to the groundwater aquifer recharge, e.g. Refs. [3,[18], [19], [20], [21], [22], [23], [24]]. Although droughts occurrences are unpredictable which make its consistent forecasting difficult, e.g. Ref. [25]. Thus, many of the research studies were focused on measurement of the risks with considerable number of them that narrowed their studies on groundwater aquifers for availability of the scanty and portable water resources.

Efforts by the United Nations (UN), member states to achieving the sustainable 2030 agenda for social and economic development purposes, has led to proper and adequate natural resources management and control, e.g. Refs. [[26], [27], [28]]. It is crucial to understand the inter relationships amongst the UN sustainable development goals (SDGs), with particular emphasis on food-climate-energy-water nexus which would enables lifelong sustainability and economic development, e.g. Refs. [[26], [27], [28]]. All the resources in the UN SDGs cannot be isolated as they are all interwoven, e.g., climate variability cannot be separated from sustainable energy, the same way sustainable food provisions cannot be achieved without hygienic portable and sustainable water for the growing global population. Each one elucidating in groups, or in part of each other, e.g. Refs. [29,30]. The inter-dependence of the SDGs involved solving numerous socio-economic and environmental problems to meet the demands of creatures and natural systems. In a bid to fighting poverty and hunger in the society of these days, adequate priority must be given to sustainable food production through the provisions of sustainable and hygienic water in all regions.

Delineation of the subsurface stratum underlain the study area using RES2-D inversion and IP, to define dry-areas and groundwater aquifer zones for the exploitation of portable and sustainable groundwater with a view of meeting clean water for agricultural, industrial, and sanitation needs of the communities living in the area. The climatic effects on the comparative changes in groundwater level is also of prime importance, especially the fluctuations in the groundwater levels occasioned by the relative climatic changes which reflects the weather conditions in the region. Although, electrical resistivity methods have been widely used to delineate severe draughts zones with the availability of some of the reports, e.g. Refs. [22,25,[31], [32], [33], [34], [35]]. Great challenges have been confronting the physical modelling of groundwater aquifers due to insufficient continuous in-situ data representing the heterogeneity aquifer systems, or longer periods of observations in many parts of the world as reported by Ref. [36].

Applications of physical parameters obtained from geophysical prospection to quantify groundwater occurrences serves as utmost significance for the provision of sustainable hygienic groundwater to meeting the agricultural, domestic, and industrial consumptions of the millions of people in the community that solely dependent on groundwater. Therefore, this study seeks to address lasting solutions to declining and scarcity of water supplies in the State of *Selangor*, through applications of integrated surface geophysical techniques with borehole in-situ data to construct the hydrogeophysical models of groundwater quality index of the area, that will help in the efficient water resource management. With availability of sustainable fresh and portable water will perhaps leads to food security and economic growth of these communities.

Ambiguities arises from surface and subsurface geophysical mappings due to data acquisition, processing and validation of results, humans and errors from geophysical instruments used, together with the subsurface stratum conditions, necessitates integration of the surface mapping techniques with in-situ borehole well logs to study the lithological units, e.g. Ref. [37]. Since observed groundwater levels within an aquifer unit are non-continuous in a given time and space, the results obtained from RES2-D and IP alone are inadequate to model the groundwater estimated volume, e.g. Refs. [33,36]. The aquifer parameters are none distributive which give rise to qualms in the data interpretations if proper care is not taken into consideration. The in-situ borehole well logs helped to overcome the uncertainties that would have arise from the estimated volume of groundwater occurrences in the study area if surface geophysical methods are applied only.

To connect effectively with the people in these communities, and facilitate water policies for adequate resource allocation, the hydrogeophysical models of groundwater quality index obtained from the results of geophysical mappings for the study area, and the boreholes well logs, serves as guides for the detail needed information on the boreholes sitting, depths to the aquifers units, and other geophysical parameters that support exploitation of clean sustainable hygienic groundwater for the community's food security and social economic stability as population of the area soars. Sustainable climate-water nexus is for the policy makers in the natural resource management to adequately plan for sustainable adaptation strategies through corrective measures that will minimize the impact of weather and climate on groundwater resources.

## 2 Material and methods

### 2.1 The study area

**Fig. 1:**
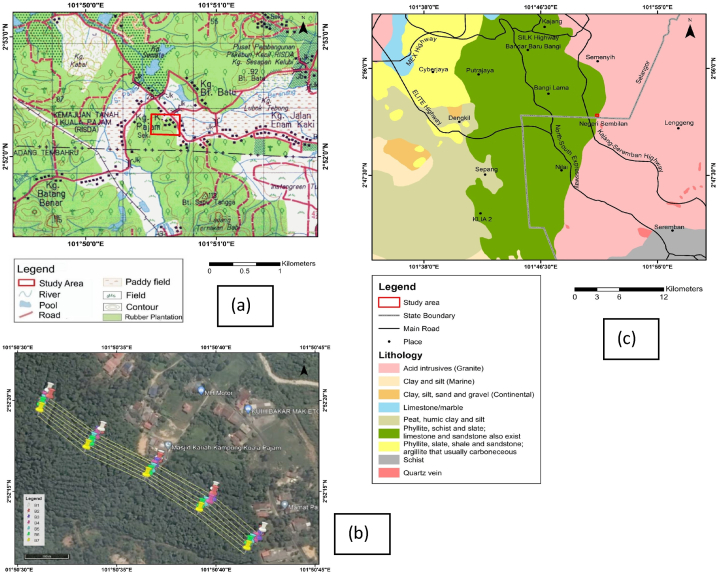
(a) Topographical map of *Selangor* showing the study area; (b) Geophysical layout showing the survey lines positions on the Google Earth satellite imagery; (c) Geology map of *Selangor* showing the study area.

Fig. 1a, showed the Topographical map that was published by *Jabatan Ukur dan Pemetaan Malaysia (JUPEM) in* 1994, and extracted from the topographic sheet number 3856, e.g., (Seremban Topographical map). The present study area is located at the hilly area in the Rubber Estate with highest elevation contour of about 55 m and could be accessed through *Jalan Enam Kaki* route.

The area is drained by rivers *Pajam* and *Beranang*. River *Pajam* is located approximately 860 m from the northwest (NW) to the study site. River *Beranang* is located approximately 250 m from the eastern side of the study area. The geophysical mapping was carried out behind the *Masjid Kampung Kuala Pajam* as shown in Fig. 1b. The selection of the study area was based on mapping groundwater potential zones in hard rock's geological terrains according to the models reported by Ref. [2].

Average annual precipitation recorded in the area was about 2500 mm, with the northeast (NE), Monsoon seasons producing the maximum peak values from months of October to January yearly which is characterized by significant seasonal variations in precipitations, temperature, evaporation, extremes wets and dryness periods. The meteorological station in *Selangor* recorded maximum temperature of about 39 ᵒC, and a minimum of about 23 ᵒC, with >96% mean relative humidity for this area. The research work being reported covered *Kampung Kuala Pajam, Beranang, Selangor* State communities. Geologically, i.e., Fig. 1c, the study area is underlain by Permian Formations that consist mainly of phyllites, slate, and shale with sandstone, siltstones, and schist as the subsidiary rock units, and a major faults trending along the NE-SW directions as delineated from the surface geophysical methods.

Geologically, *Kampung Kuala Pajam* is located at the *Kenny* Hill Formation as shown in Fig. 1a. The possible age of the formation was reported to be of late Paleazoic; Carboneferous by Ref. [38]. The formation consists of sequence layering of shale, siltstones, and sandstones. Shale and siltstone have metamorphosed into phyllite (e.g., Fig. 1c), and shows some presence of foliations and cleavages that are not parallel to the bedding planes. Generally, the sandstones are much richer in quartz, with the abundance of quartz veins observed at the study site (e.g., Fig. 1c). Some of the Outcrops observed at the Kenny Hill Formation were also found around Kuala Lumpur, and *Petaling Jaya* areas, e.g. Ref. [39]. The same formation units were also located at a bigger area within *Kajang* and *Bangi* environments. Some of the phyllite outcrops has been exposed as observed near the study site. The *Kenny* Hill Formation is part of the orogeny Formations that consists of various types of rock units with varieties of origins.

### 2.2 Method of data acquisition

**Fig. 2:**
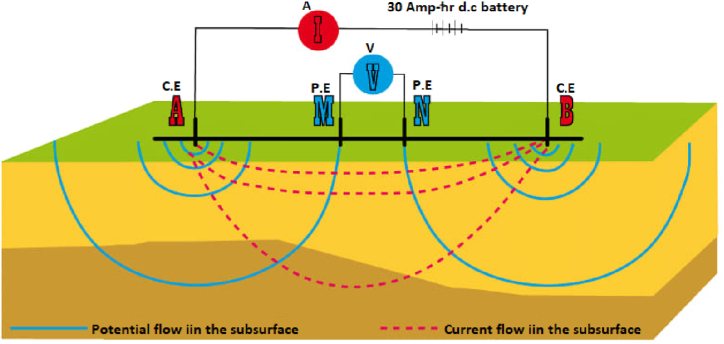
Schematic diagram for the Wenner-Schlumberger (W–S) RES2D ERT showing the d.c source and metal electrode connectorrs labelled C.E and P.E.

Geophysical field data acquisition was carried out using ABEM SAB4000 Terrameter land imaging equipment in combination with auto selection ABEM ES 10–64 unit. The RES2-D ERT surveys were accomplished at 5 m electrode constant spacing, using 61 metal steel electrodes via two reels of multi-core cables [37,40]. A microcomputer unit was incorporated to the ABEM ES 10–64 selector as a switcher unit, which automatically pick-out four energized electrodes promptly, was used for each measurement. Each of the RES2D ERT surveyed profile lines, was made up of a single electrode spread of 400 m, with the application of pole-dipole electrodes array technique. The two dimessional resistivity inversion (RES2-DINV) software, by Ref. [41], was applied to model the recorded resistivity field datasets obtained from *Beranang, Selangor* community. The data acquired necessitates a qualitative approach to using a 12 V, 30 Amp-hr d.c battery as the source of current (A), injection to the subsurface strata, through the metal steel electrodes at points A & B, (e.g., current electrodes C.E). The consequential potential difference (V), dropped across these metal steel electrodes at points M & N, (e.g., potential electrodes P.E), are subsequently measured at other electrodes positions in the regions of the current flowing through them using parallel connections, e.g., Fig. 2.

Observations with subsurface structural layers are better mapped using the electrode cable geometry together with a switching cycle that defined the address and protocols files which allows user-defined arrays and survey approach. For this work, the Wenner-Schlumberger (W–S), configurations were selected. On the other hands, irregular heterogeneity in the subsurface structural layers is better delineated adequately with the application of the Pole-Dipole (PD) configurations. A combination of both W–S and PD will always produce better results. The PD array with the W–S configuration were selected for the data acquisition, because of their influence on the RES2-D inversion model applied. The combination has comparatively high-quality regular coverage, and higher signal to noise strengths' ratio. The model helped to eliminate ambiguities from the recorded field data, and much less sensitive to telluric noise than the pole-pole array, e.g. Refs. [1,21,22].

The pole-dipole array has four collinear electrodes. The second current electrodes (e.g., C2), was positioned at an “effective infinite” distance that must not be paralleled to the survey line, at approximately five times larger than the distance of C1 and P1. The other current electrode (C1) was positioned in the vicinity of the two potential electrodes (P1 and P2). This geometry was used because of its higher signal strengths compare to the dipole-dipole configurations. Thus, the geometry will generate higher depths of investigation. Besides, the less exposure to telluric noise datasets produces from these electrode arrays as both the potential electrodes are positioned according to the survey lines.

### 2.3 RES2-D ERT and IP data processing technique

**Fig. 3:**
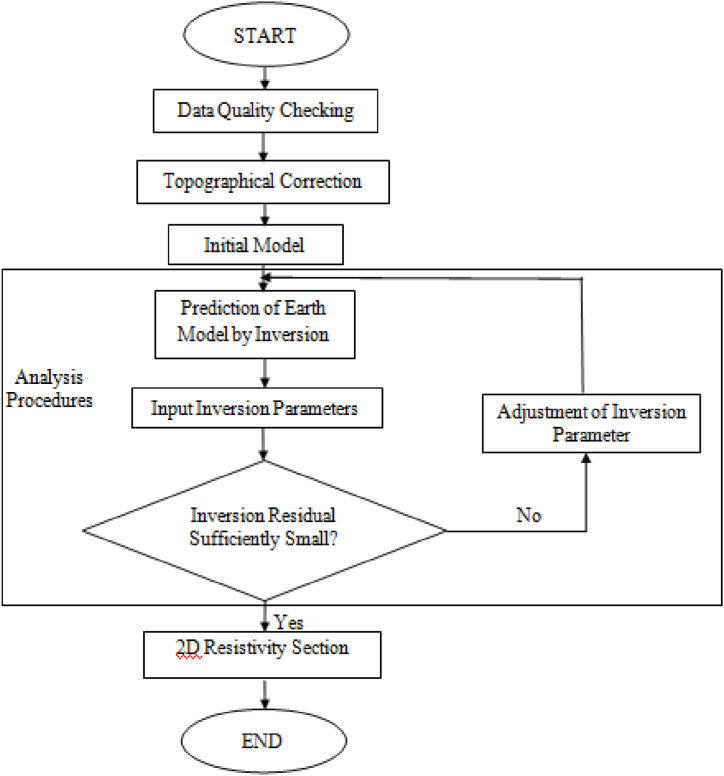
Step by step RES2-D and IP data processing flow chart. Adopted from Ref. [37].

The raw data were collected and transferred to the computer using the software for SAB4000 Utilities and *DAT format for data processing with the RES2-DINV software. SAB4000 software also has a presetting for the exchanges of data file formats, e.g., *.B4K to some other format similar to *REX, *.LAS, *.AMP, *.RPD, *.REX, and *DAT. Data files that have been transferred to the *DAT format is processed using RES2-DINV software to generate the RES2-D profiles. The step-by-step processing of the field data is presented in Fig. 3.

Data quality check was carried out as soon as the data files transfer is completed. The topography data of the area was checked for correctness before generation of the initial RES2-D model. Data from RES2-D profiles are then processed using RockWorks software to generate and produced a 3-D model. Prior to the production of 3-D model, the recorded field data in excel file format. i.e., (.xlsx) was imported into the software first. The field data files contain data for distances in metres of the survey points (X), spacing between the survey lines (Y), elevation (Z), and resistivity values in Ω-m, and the chargeability in ms. These datasets were therefore processed into solid model of the 3-D generated for the study area.

## 3 Results and discussions

### 3.1 RES2-D and IP profiles along survey line B1

**Fig. 4:**
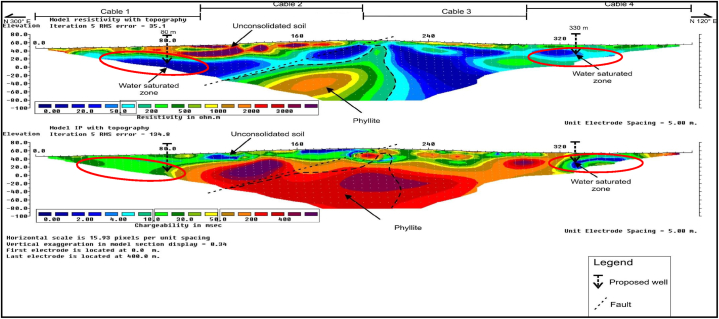
RES2-D and IP profile along survey line B1.

RES2-D and IP profiles along survey line B1 is presented in Fig. 4, with approximate line orientation in the N 300 °E direction, and a total survey length of 400 m, at inter electrode spacing of 5 m. The subsurface strata resistivity values recorded varied between about 0 Ω-m and 4000 Ω-m. Three major lithologic units were clearly delineated in the interpreted RES2-D data as; (i) the groundwater saturated zone/aquifer zone, (ii) the unconsolidated soil, and (iii) the weathered bedrock comprises mainly of phyllites rock formation. The lowest resistivity, and chargeability values respectively varied from 0 to 100 Ω-m, and 0–30 ms. This unit was interpreted as the groundwater saturated zone, located at about 20–80 m along the survey line, at depth of about 20–80 m. The highest resistivity values of 500–2000 Ω-m, was interpreted as the phyllite bedrock which is located at an approximate depth of about 20–140 m.

The RES2-D and IP profiles results showed delineation of faults with NE-SW orientations at about 210 m, along the horizontal distance of the survey line. Two borehole positions were pinpoints at 80 m, and 330 m respectively, with a view to salvage the community sustainable water needs.

### 3.2 RES2-D and IP profiles along survey line B2

**Fig. 5:**
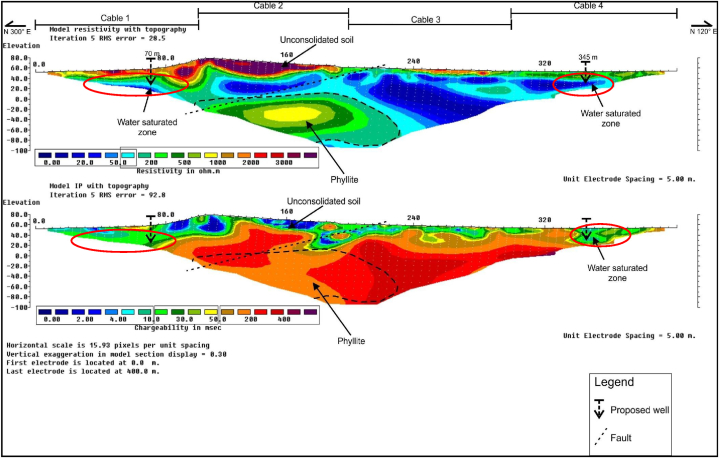
RES2-D and IP profile along survey line B2.

Resistivity and Induced Polarization profiles along survey line B2, is as shown in Fig. 5. The lowest RES2-D value varied from 0 to 100 Ω-m, that was delineated as water saturated zone/aquifer unit. Low chargeability value, i.e., 4–20 ms, was recorded for the zone, at depths of about 30–60 m. The highest resistivity and chargeability values respectively varied from about 100 to 1000 Ω-m, and 50–300 ms. The weathered interpreted phyllite bedrock unit was delineated at horizontal distance of between about 110 and 230 m, along the survey line.

From the RES2-D and IP profiles, the faults zone was delineated along Northeast-Southwest directions at horizontal distance of about 220 m.

### 3.3 RES2-D and IP profiles along survey line B3

**Fig. 6:**
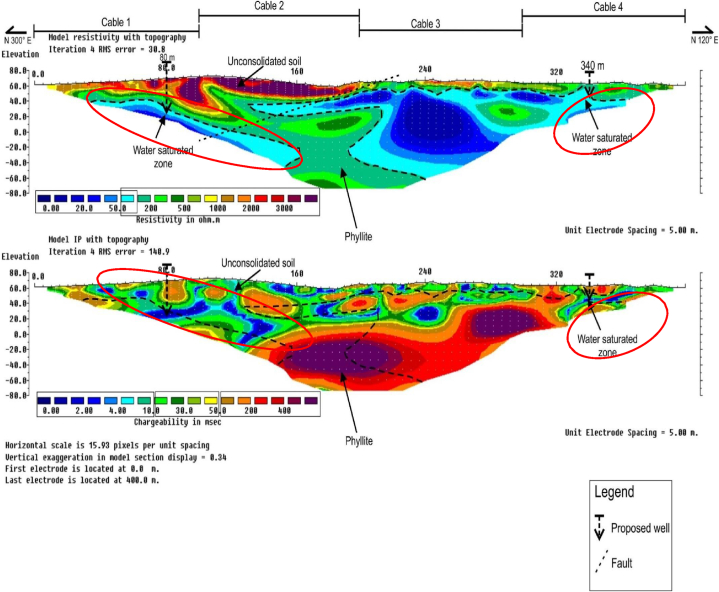
RES2-D and IP profile along survey line B3.

The RES2-D and IP profiles along survey line B3 is presented in Fig. 6. The lowest resistivity and chargeability values recorded were 0–100 Ω-m, and 0–30 ms, respectively. Interpreted as water saturated/aquifer zones between about 45 and 130 m, and 320–360 m. along the survey line. The water saturated/aquifer zone were delineated at depths of between about 40 and 80 m, and 20–60 m respectively. The highest recorded resistivity values of about 100–500 Ω-m were located at about 150–240 m, along the survey line. The unit was interpreted as the weathered phyllite bedrock with high chargeability values of between 50 and 500 ms.

From the RES2-D and IP profiles, a faults zone with Northeast-Southwest orientation, was delineated at about 215 m along the survey line. Based on the results obtained from both the low resistivity and chargeability values, two borehole drilling locations were proposed at points 80 m, and 340 m, along the survey line which would serves the community water requirements.

### 3.4 RES2-D and IP profiles along survey line B4

**Fig. 7:**
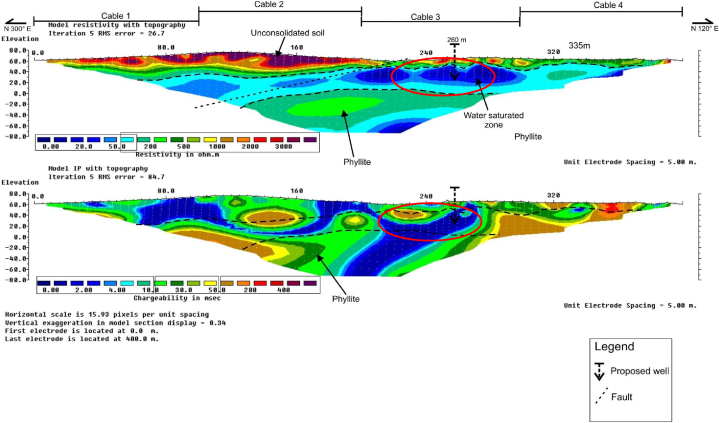
RES2-D and IP profile along survey line B4.

Fig. 7, shows the RES2-D and IP profiles along the survey line B4. Zones of water saturated/aquifer were delineated at approximate distance of about 210–275 m along the survey line with depths of between about 25 and 50 m. Low resistivity and chargeability values were respectively recorded as, 0–100 Ω-m, and 0–10 ms for this unit at horizontal distance of between about 120 and 280 m, and depths of between 60 and 110 m. The weathered phyllite bedrock unit with moderately high resistivity and chargeability values of between 20 and 500 Ω-m, and 50–100 ms, were delineated at horizontal distance of between about 120 and 280 m, at an appropriate depth of between 60 and 110 m.

Faults zone was delineated along the Northeast-Southwest directions at about 230 m along the horizontal distance of the survey line. Based on both the RES2-D, and IP profiles results, borehole drilling position was pinpointed at about 260 m, along the survey line to supply the needed portable water for the community on the basis of the geoelectric parameters recorded.

### 3.5 RES2-D and IP profiles along survey line B5

**Fig. 8:**
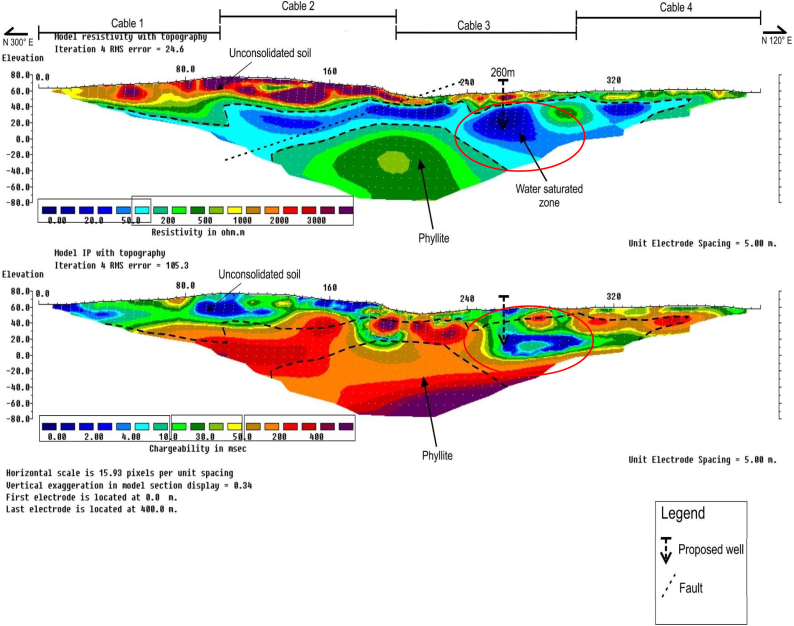
RES2-D and IP profile along survey line B5.

Fig. 8, showed the RES2-D and IP profiles along survey line B5. Aquifer/water saturated unit was delineated at approximate distance of between and 250–330 m, along the survey line with depths of about 20–70 m. The unit is characterized by respectively low resistivity and chargeability values, typically of between 0 and 200 Ω-m, and 0–20 ms. Phyllite weathered bedrock unit was delineated at horizontal distance of between 125 and 260 m, with approximate depth of about 55–140 m, along the survey line. The bedrock unit has high recorded resistivity values of between about 500 and 750 Ω-m, and a corresponding high chargeability value of between 50 and 500 ms.

Based on the two profiles, the NE-SW trending faults zone was delineated at about 210 m. From the results obtained, borehole drilling location was pinpoint at 260 m along the survey line for the groundwater exploration that would serve the community.

### 3.6 RES2-D and IP profiles along survey line B6

**Fig. 9:**
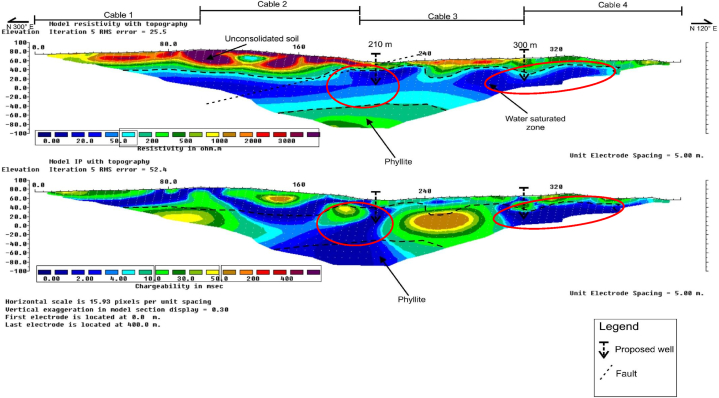
RES2-D and IP profile along survey line B6.

Line B6, i.e., Fig. 9, showed the delineated groundwater saturated/aquifer unit with RES2-D and IP geophysical methods given varied geoelectric parameters values of between about 0 and 100 Ω-m, 0–10 ms, and 0–30 ms, respectively, at approximate horizontal distances of between 120 and 230 m, and 205–360 m, along the survey line. Depths to the groundwater saturated/aquifer unit was delineated between 30 and 80 m, and also 20–90 m. At the point between horizontal distance of about 135–270 m, along the survey line, was interpreted as the weathered phyllite bedrock unit with varied depths of between 80 and 140 m. Moderately high resistivity values of between 500 and 750 Ω-m, and a corresponding chargeability value of between 0 and 30 ms were recorded for the weathered unit.

The RES2-D and IP geoelectric parameters was able to delineate the NE-SW faults at about 220 m along the horizontal distance. Two proposed borehole locations for groundwater drilling were respectively pinpoint at 210 m, and 300 m along the survey line.

### 3.7 RES2-D and IP profiles along survey line B7

**Fig. 10:**
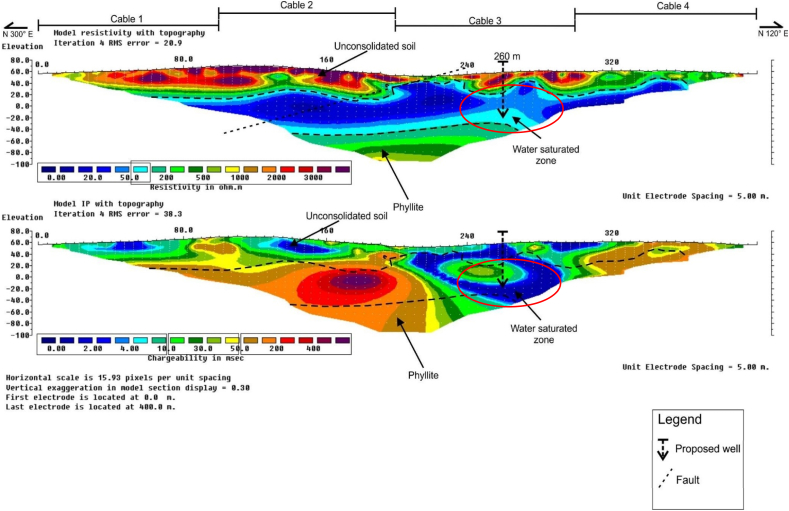
RES2-D and IP profile along survey line B7.

Fig. 10, showed RES2-D and IP geoelectric parameters for the survey line B7. Low resistivity, i.e., 0-100 Ω-m, and the corresponding chargeability, i.e., 0–30 ms, were respectively delineated at horizontal distance of about 210–310 m, along the survey line. The depths of between 20 and 50 m, was interpreted as the groundwater saturated/aquifer zone. The weathered phyllite bedrock unit was delineated along horizontal distance of between 140 and 265 m, on the survey line with varied depths of between 80 and 140 m. Moderately high resistivity of between about 500 and 2000 Ω-m, and the corresponding chargeability of between 50 and 200 ms were recorded for the unit as interpreted.

The NE-SW trending faults zone was delineated at about 220 m. Based on the results obtained, borehole drilling location was pinpoint for groundwater exploration at 260 m along the survey line with probable depth of about 25–50 m that would alleviate the challenges facing the people in the community.

### 3.8 Hydrogeophysical modelling of the RES2-D and IP results with the borehole well logs

**Fig. 11:**
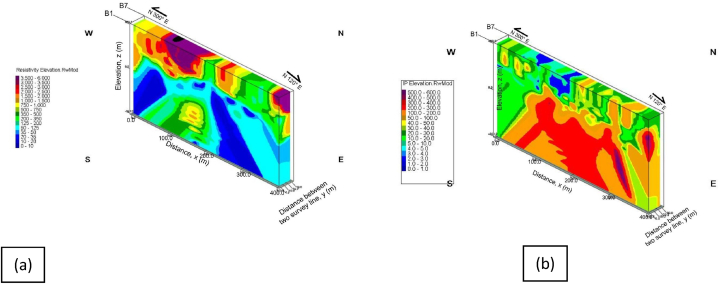
3-D hydrogeophysical models of the subsurface units generated from; (a) RES2-D, and (b) IP geoelectric results, with the borehole well logs.

**Fig. 12:**
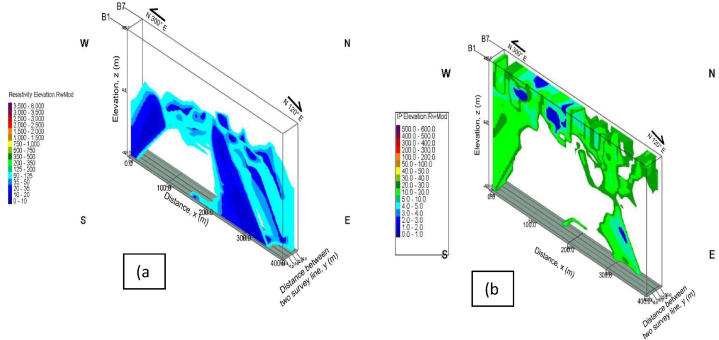
3-D hydrogeophysical models of the groundwater aquifer unit extracted from; (a) RES2-D, and (b) IP geoelectric results, with the borehole well logs.

The primary focus of this work is to model subsurface zones saturated with groundwater accumulations for borehole sitting that would serve the community's sustainable hygienic water needs on the basis of the geoelectric parameters recorded in Table 1. Three dimensional (3-D), hydrogeophysical models of the results from RES2-D and IP geoelectric, was constructed to quantify volume of the groundwater occurrences at the study area as presented in Fig. 11, Fig. 12. The models were generated through the combinations of the geoelectric results from all the survey lines at inter-profile distance of 5 m. However, Fig. 12, showed the groundwater potential/aquifer zone interpreted based on the recorded resistivity values of <125 Ω-m, and chargeability values of between 0 and 30 ms. The units were delineated between 100 and 200 m, for the first segment, and from 300 to 400 m, in the second segment, at varied depths of between 30 and 100 m. Volume of the groundwater accumulations residing within the subsurface reservoir of the study area was then estimated to be about 2.86 Mega CBM.

**Table 1:** Summary of the groundwater/aquifer saturated zones and proposed borehole drilling location along the survey lines.

Survey lines	Distance along survey line (m)	Depth (m)	Resistivity (Ω-m)	Chargeability (ms)	Propose borehole drilling point (m)
B1	20–80	20–80	0–100	10–30	80
	330–360	20–60	0–100	0–20	330
B2	30–90	30–60	0–100	4–20	70
	330–360	30–60	0–100	5–20	345
B3	45–130	40–80	0–100	0–30	80
	320–360	20–60	20–100	0–40	340
B4	210–275	25–50	0–100	0–10	260
B5	250–330	20–70	0–200	0–20	260
B6	120–230	30–80	0–100	0–30	210
	205–360	20–90	0–100	0–10	300
B7	210–310	15–50	0–100	0–30	260

Two validating boreholes wells were drilled along the horizontal distance of 210 m, on the survey line profile B1, and 260 m, on the survey line profile B7 respectively, i.e., Table 2, Table 3. The detailed borehole log results, (i.e., Appendices A1 and A2), showed good correlations between the resistivity profiles results and the borehole well logs data. Wet silty-gravely-sand zones from the depths of between about 10 and 39 m, and the weathered phyllite bedrock unit was delineated at about 39–97 m, as shown on the first validation borehole. Groundwater yields was extracted until depth to about 97 m. The consequences of groundwater quality deterioration occasion by deeper boreholes in regions closer to coastal areas that could rendered groundwater unsustainable were carefully observed. The geopotential height of the study area, the borehole pumping pressure and the study site distance from the coastal sores provided the needed advantages to overcome challenges of saltwater intrusions that could rendered the groundwater unsuitable for agricultural, domestic, and industrial consumptions.

**Table 2:** Validation borehole well log data at point 210 m along the survey line profile B1.

Borehole Location at: Beranang, Selangor. N 427672, E 317740		Drilling Method: Air Percussion Well Type: Deep Bored
Total Depth: 97.0 m		
Depth (m) 0	Lithologic Description	
	Sandy Silt	Silt Sand Gravel Phyllite
0–6	Silty Sand	
6–9	Silty Sand	
9–15	Silty Sand	
15–24	Silty fine sand with some gravels.	
24–39	Silty gravel.	
39–55	Phyllite with quartz grains (veins).	
55–73	Phyllite with quartz grains(veins).	
73–97	Phyllite with some quartz grains(veins).

**Table 3:** Validating borehole well log data at point 260 m distance along survey line B7.

Location at: Beranang, Selangor. N 427635, and E 317673		Drilling Method: Air Percussion Well Type: Deep Bored
Total Depth: 55.0 m		
Depth (m)	Lithology Description	
0	Sandy Silt	Sandy Silt Sand Gravel Phyllite
0–3	Sandy Silt	
3–6	Brownish Orange, Sandy Silt (Residual Soil)	
6–8	Sandy Silt	
8–10	Fine Sandy Silt	
10–16	Silty Sand	
16–18	Silty Gravels	
18–25	Silty Sand with some gravels and pebbles.	
25–31	Phyllite with quartz grains (veins).	
31–37	Phyllite with quartz grains (veins).	
37–43	Phyllite with quartz veins.	
43–49		
49–55	Phyllite with quartz veins.	

The top/unconsolidated soil filled up the first lithologic unit from the ground surface down to about 9 m, Though, grains of weathered phyllite rock formation with other rocks such as silty sands were delineated from about 9 m downwards.

The second validating borehole well was drilled at point 260 m, along the survey line profile B7, with a maximum depth of about 55 m. The well log data from the borehole lithologic sequence showed correlated results with that of the resistivity and IP surveys. Depths from between about 10 m and 55 m, below the ground surface, produced wet aquifer zones. Depths of between 18 m and 49 m, was filled with gravel, quartz, and phylite pebbles with increase in sizes as the depth of the borehole increases but it has no effect on the groundwater yield, as it increases as the depth of the borehole increased.

## 4 Conclusion

In planning sustainable adaptation strategies for growing population of the state of *Selangor*, Peninsular Malaysia, the role of groundwater resources in ensuring food security, socio-economic development, and societal wellbeing, is critical in the face of unpredictable climate variables and droughts. Many research proposed measures to correct or minimized the impacts of climate variability and droughts on water resources, e.g. Ref. [25]. However, a forward-looking approach intended to considerably reduce the human vulnerability to the future risks of exposures that could threatened socio-economic development, food production and other societal sectors is crucial.

The results generated from the seven RES2-D and IP profiles geoelectric parameters together with the borehole logs data considered, clearly delineated water saturated aquifer units as presented in Table 1, and the models generated in Fig. 11, Fig. 12 which helped in the estimation of groundwater reservoirs. Based on the borehole logs data, the subsurface geology of the study area was shown to be by and large, mostly shales, and siltstones that metamorphosed into phyllite rock units that sustained groundwater aquifers in the area which will help water resources policy management and authority to enable greater resilience and sustainability irrespective of the magnitude of future droughts.

The model generated from our results showed availability of groundwater resources that would meet the demands of the communities irrespective of the future climate and weather scenarios. The RES2-D and IP parameters recorded also showed great promising aquifers yields in the weathered phyllite hard rock formations to meet the diverse demands without fear of experiencing supplies deficits as the demands soars within and outside sustainable livelihoods. In all the seven survey lines executed, the major faults trending along NE-SW directions was delineated. The geoelectric results obtained from the survey lines B4, B6, and B7, produced abundance subsistence groundwater occurrences capable of serving the community's sustainable domestic and agricultural water needs irrespective of the pumping rates with no significant impact on the ecosystems and the environment for future planning and adaptation strategies. The modeled results produced potential bedrock deformations along these three profile lines with deep cracks/fissures, and weathered bedrock with displaced subsurface hard rocks that formed groundwater aquifers units at depths >50 m.

Though, the interpreted validation borehole well logs data from the two validating monitoring wells provides same rock type at various depths, the differential in their resistivity and chargeability values may not be unconnected to the influence of fractured bedrocks, the degree of weathering, saturated formation water, and the mineral contents on the resistivity and IP of the subsurface rocks, e.g. Ref. [40]. Based on the results from the two-validation borehole well logs data; RES2-D and IP profiles, together with the modeled 3-D generated, it could be therefore concluded that.

• The groundwater saturated/aquifer zones were clearly delineated as confirmed by the correlations of the results that could leads to food security and economic growth of these communities.
• The phyllite outcrop sited at the vicinity of the study area, together with the two validating boreholes well logs, provided evidence of the delineated weathered phyllite bedrock units underlain the area to produce great quantity of subsistence groundwater capable of serving the communities.
• The delineated NE-SW trending faults along all the survey lines, could be the conduit water ways to recharge the aquifer units even though hard rock aquifers present small buffer storage. This is significant to the aquifer recovery during prolong future drought episode as the impact will be minimal or no effect.
• Saturated groundwater reservoir estimated volume of about 2.86 Mega CBM within the subsurface of the study area, would be a long-lasting solution to the perennial acute water shortages confronting the community during prolong drought seasons as it provides an acceptable guide and realistic approach to planning for sustainable hygienic water supplies by the decision makers.

The success from 3-D hydrogeophysical modeling of groundwater aquifer units with the applications of borehole well logs data, RES2-D and IP profiles, resides on the support of climate-water nexus sustainability, looking from the volume of groundwater produced through the geophysical results, and the two validated borehole well logs data to meet the *Beranang, Selangor*, Malaysia, daily portable hygienic domestic water usage, irrespective of the prevailing climatic conditions. Although, the consequences of groundwater quality deterioration occasion by deeper boreholes in regions nearer to coastal areas was taken into consideration while drilling the two validated borehole wells. The maximum depth of the first well at < 100 m is much shallower to creating source of concerns on aquifer degradation from saltwater intrusions that could lead to unsustainable groundwater extraction. Long-term sustainable clean and hygienic groundwater implementation across the Peninsular Malaysian states could put an end to acute water shortages that bring about disruptions to foods and industrial production. Our approach is devoid of any consequential impacts from groundwater depletion.

To transform groundwater resources towards achieving improved, and sustainable resilience irrespective of the magnitude of future climatic conditions involved commitment by all sectors and players in the management of water resources. Therefore, it is a call for action by the political office holders, decision makers, public and private sectors practitioners, scientific community, and the society in general to jointly build a sustainable environment for the wellbeing of all.

## Author contribution statement

John Stephen Kayode: Conceived and designed the experiments; Performed the experiments; Contributed reagents, materials, analysis tools or data; Wrote the paper.

Mohd Hariri Arifin: Conceived and designed the experiments; Performed the experiments; Analyzed and interpreted the data; Contributed reagents, materials, analysis tools or data.

Mohd Idham Mansor & Nurul Nadia Abdul Malek: Performed the experiments; Analyzed and interpreted the data; Contributed reagents, materials, analysis tools or data.

Roziah Che Musa: Sabrina Shahri & Nur Hazwani Izehar: Performed the experiments; Analyzed and interpreted the data.

Mohd Rozi Umor: Contributed reagents, materials, analysis tools or data.

## Funding statement

This research did not receive any specific grant from funding agencies in the public, commercial, or not-for-profit sectors.

## Data availability statement

Data will be made available on request.

## Declaration of interest’s statement

The authors declare no competing interests.

## Figures and Tables

**Table A.1:** Borehole well log data located at point 210 m, along the survey line profile B1.

Location: Beranang, Selangor, Malaysia. N 427672, E 317740		Drilling Method: Air Percussion Well Type: Deep Bored	
Driller's Name: Zaidi		Total Depth: 97.0 m	
Depth (m)	Resistivity(Ω-m)	Lithologic Description	Depth of aquifer and flow rate
0	100–200	Topsoil, pale brown to medium brown, sandy silt with gravels and organic materials	
0–6	100–200	Brownish orange to pale brown, silty sand with some gravels (residual soil)	Wet
6–9	100–200	Grey to pale grey, silty sand with some gravels (residual soil)	Wet
9–15	100–300	Dark grey, silty sand with some gravel. Gravel is phyllite and quartz grains	Moist/Wet
15–24	100–300	Medium grey, silty fine sand with some gravels. Gravels is phyllite and quartz grains. Grain size 2–4 mm.	Dry
24–39	100–200	Dark grey, silty gravel. Gravel is phyllite and quartz grain. Grain size 2–30 mm. Chlorite deposited at quartz grains. Pyrite minerals also presence.	Establish yield: 0.5–1 cm Wet
39–55	100–300	Dark grey, carbonaceous phyllite with quartz grains (veins). Grains size is 2–40 mm. Chloride and iron deposited in quartz grains. Pyrite minerals also presence.	Establish yield: 0.5–2 cm Bedrock established at depth 45.0 m.Wet
55–73	200–500	Medium to dark grey, phyllite with quartz grains(veins). Grain size 20–30 mm. Presence of chloride, iron ore and pyrite minerals.	Dry to moist
73–97	100–300	Dar grey, phyllite with some quartz grains(veins). Grain size 2–20 mm. presence of chloride and pyrite minerals.	Establish yield: 0.5 cm Wet

**Table A.2:** Borehole well log data located at 260 m on the survey line profile B7.

Location: Beranang, Selangor, Malaysia.N 427635, E 317673		Drilling Method: Air Percussion Well Type: Deep Bored	
Driller's Name: Zaidi		Total Depth: 55.0 m	
Depth (m)	Resistivity (Ω–m)	Lithology Description	Depth of aquifer and flow rate
0	1000	Topsoil, pale brownish yellow to yellowish orange, sandy silt with organic materials	
0–3	1000–2000	Pale brownish yellow to pale yellowish orange, sandy silt (residual soil)	Moist
3–6	1000–2000	Brownish orange, sandy silt (residual soil)	Dry
6–8	2000–3000	Pale brown, sandy silt (residual soil)	Dry
8–10	1000–2000	Light brown, fine sandy silt (residual soil)	Dry
10–16	500–1000	Dark grey, silty sand with some gravels. Gravels is phyllite and quartz grains	Wet
16–18	500–1000	Medium to pale brown, silty gravels with some pebbles of metasediment and quartz	Wet
18–25	200–500	Dark grey, silty sand with some gravels and pebbles. Gravel/pebbles is carbonaceous phyllite and quartz grains with size 2–30 mm. Quartz is from quartz veins	Establish yield: 2–4 cm Fractured zone Wet
25–31	200–500	Dark grey, carbonaceous phyllite with quartz grains (veins). Grain size is from 2 to 50 mm. chlorite ion is deposited along fractured of quartz grains. Presence of pyrite minerals	Establish yield: 2–4 cm Fractured zone Wet
31–37	100–200	Dark grey, carbonaceous phyllite with quartz grains (veins). Grain size is from 2 to 50 mm. Mineral presence. iron oxide, pyrite, and chlorite.	Establish yield: 2–5 cm Fractured zoneWet
37–43	20–50	Dark grey, carbonaceous phyllite with quartz veins. Grain size is 2–70 mm. Chlorite presence in quartz veins and also, presence of iron oxide and pyrite minerals	Establish yield 5–8 cm Highly fractured zone Wet
43–49	20–50		
49–55	20–50	Dark grey carbonaceous phyllite with quartz veins. Quartz veins size is 2–20 mm	Wet
